# Young fatherhood, masculinities, and structural factors in South Africa

**DOI:** 10.3389/fsoc.2024.1410801

**Published:** 2024-07-25

**Authors:** Tawanda Makusha

**Affiliations:** Public Health, Societies and Belonging Division, Human Sciences Research Council, Durban, South Africa

**Keywords:** young fatherhood, masculinities, structural factors, father involvement, South Africa

## Abstract

**Background:**

This paper explores the intricate interplay between young fatherhood, masculinities, and structural factors in South Africa. The country grapples with a complex web of social, economic, and cultural dynamics that shape the experiences of young men’s transition into fatherhood.

**Methods:**

This qualitative study used snowball and purposive sampling techniques. In-depth interviews were undertaken with 24 young biological fathers aged between 18 and 24 years in an informal settlement in Durban (12) and a rural community in Pietermaritzburg (12), KwaZulu-Natal. Four focus-group discussions, in groups of four participants, were conducted with 16 of the participants who took part in the in-depth interviews. Data were analysed thematically on the local constructions of young fatherhood and masculinities and how both were associated with structural factors.

**Results and discussion:**

Young fatherhood in South Africa is associated with a number of structural vulnerabilities, such as living in communities with high alcohol and drug abuse, low educational attainment, inadequate access to healthcare, unemployment, poverty, and crime. These structural vulnerabilities, deeply entrenched in the country’s history and socioeconomic fabric, together with prevailing notions of masculinities, often rooted in hegemonic ideals of dominance and control, intersect with societal expectations of fatherhood, thereby shaping young men’s identities, roles, and responsibilities as fathers.

**Conclusion:**

The findings suggest young men’s involvement during the transition to fatherhood appears multi-determined. To effectively support young fathers and promote family well-being, it is imperative to address the root causes of structural inequalities, challenge rigid norms of masculinities, and foster inclusive policies and programmes that empower young men to embrace their roles as caregivers and agents of change within their families and communities.

## Introduction

The past three decades have witnessed a growing literature on the importance of fathers to the development of their children and a considerable shift in social expectations associated with the role of fathers (Morrell, 2006; Hosegood and Madhavan, 2012; Crespi and Ruspini, 2015; Makusha and Richter, 2018). However, despite this evolution, South Africa, like many other countries, still grapples with the complex intersection of structural factors, masculinities, and young fatherhood. This paper delves into the multifaceted dynamics shaping the experiences of young men as they navigate the transition to fatherhood in the South African context. Fatherhood is linked to manhood, which is associated with masculinities, while both fatherhood and masculinities are associated with structural factors in South Africa (Morrell, 2005; Morrell, 2006; Hendricks et al., 2010; Enderstein and Boonzaier, 2015). Navigating the transition to fatherhood at a young age presents unique opportunities and challenges for South African men. While fatherhood can be a source of pride and identity formation, it also entails significant responsibilities and sacrifices, particularly in contexts marked by structural inequalities and limited support systems.

In South Africa, young fathers must negotiate competing demands of education, employment, and family obligations – including being a provider, protector, advisor, caregiver, and nurturer, often without adequate resources or social support networks (Swartz et al., 2013; Clark et al., 2015). In this context, balancing relationships with responsibilities and anxieties with the pleasures of childrearing challenges young fathers’ identities, relationships with their partners and families, and their sense of self as competent adults (Enderstein and Boonzaier, 2015).

Central to the experiences of young fathers in South Africa are prevailing norms of masculinities. Traditional constructions of masculinities, often characterised by traits of dominance, toughness, and emotional stoicism, intersect with societal expectations of fatherhood, shaping young men’s identities and roles as fathers (Macht, 2020). Hegemonic ideals of masculinity, rooted in patriarchal structures, reinforce the notion of men as providers and protectors, thereby influencing young fathers’ perceptions of their responsibilities and agency within their families (Sikweyiya et al., 2017). However, these rigid norms of masculinities can also be constraining, limiting young men’s ability to express vulnerability, seek support, or engage in caregiving responsibilities traditionally associated with motherhood (Chikovore et al., 2016). Moreover, societal expectations of male sexualities and masculinities may intersect with early fatherhood, perpetuating cycles of unplanned pregnancies and non-resident fatherhood.

A critical lens through which to understand young fatherhood in South Africa is the backdrop of structural factors. Historically rooted in apartheid policies, these factors continue to perpetuate inequalities along racial, socioeconomic, and geographic lines. Poverty, unemployment, and inadequate access to education, healthcare, and housing disproportionately affect marginalised communities, exacerbating the vulnerabilities faced by young men entering fatherhood. The legacy of apartheid also manifests in intergenerational trauma, further complicating the social landscape for young fathers and their families.

Unemployment rates, particularly among young men, remain alarmingly high, limiting economic opportunities (Statistics South Africa, 2024) and perpetuating cycles of poverty (Ward et al., 2015). This economic disenfranchisement not only affects material well-being but also shapes perceptions of self-worth and societal roles. In such contexts, traditional markers of masculinities tied to providing for one’s family may become unattainable, leading to feelings of emasculation and disempowerment among young fathers.

## Methods

### Study data and sample

This study on “*What makes a man and fatherhood in South Africa*” was conducted between October 2022 and April 2023. To avoid leaving out eligible participants and address the theoretical issues of the ‘invisibility of young fathers’, the study utilised purposive and snowball sampling techniques targeting young biological fathers aged 18 and 24 years in the study communities. In-depth interviews were conducted with 24 young Black biological fathers in an informal settlement in Durban (12) and a rural community in Pietermaritzburg (12), KwaZulu-Natal. Four focus group discussions (in groups of four) were undertaken with 16 of the 24 young Black biological fathers who took part in the in-depth interviews. Interviews were conducted either at the participants’ homes or community halls, while all focus group discussions were conducted at the community halls in Durban and Pietermaritzburg.

The interview and focus group discussion guides included the sociodemographic information of participants, their views on what makes a man in South Africa, their understanding of what makes a father and fatherhood in South Africa, their reaction to the pregnancy of the mothers of their children, their roles and responsibilities as fathers, the living arrangements with their children, and how structural factors such as education, health, poverty, and unemployment impact their roles as fathers.

In-depth interviews took between 45 and 60 min, while focus-group discussions took between 90 and 120 min. All the in-depth interviews and focus-group discussions were conducted in Zulu and digitally recorded with the permission of the respondents. Participants were encouraged to adhere strictly to confidentiality, anonymity, and privacy. Participants were also informed that their identities would not be disclosed and pseudonyms would be used in both the analysis and write-up of the study findings.

### Data analysis

The interviews were transcribed and translated from Zulu to English. This was followed by thematic data analysis, involving the coding process guided by research questions and the existing literature using qualitative analysis software, NVIVO 13. The coding process determined the local constructions of young fatherhood and masculinities and how both were associated with structural factors.

### Ethical approval

The study protocol was approved by the Human Sciences Research Council’s Research Ethics Committee (REC: 5/19/08/22). Informed consent was sought from all participants. Additionally, permission to conduct the study was sought from community gatekeepers in the two communities, including community and traditional leaders.

## Results

The data examined focused on the sociodemographic characteristics of the participants, young fatherhood, and what makes a man in South Africa – particularly focusing on the different forms of masculinities and the impact of structural factors on their roles and responsibilities as fathers.

### Sociodemographic characteristics

All 24 participants were unmarried and did not live with their children. Three reported being in romantic relationships with the mothers of their children, while the rest reported that they had separated from the mothers of their children. Seven of the 24 young fathers in the study had a matric qualification, while the rest did not complete matric. Nine young fathers were formally employed, while the rest indicated that they relied on precarious employment opportunities.

### Narratives of young fatherhood

#### Readiness

This theme focused on the experiences of young fathers as co-parents to their children. While the majority highlighted that at the time of pregnancy and the birth of their children, they were not ready to become fathers, a few indicated that they were ready to be fathers. One participant highlighted:

When the mother of my child told me that she was pregnant, I panicked, and I was scared. I did not know what to say because I was not ready to be a father. At that time, I was only 17 years old. I felt I had made a huge mistake. I did not know what to tell my mother because we were already struggling financially at home, and bringing a child home was an added responsibility for my mother, who is also unemployed and relies on social grants (Skhaleni, 18 years old).

The majority of the participants shared Skhaleni’s story. Most of these young fathers spoke about the economic challenges they were already facing and the difficulties they anticipated in being involved fathers without the ability to provide financially. These young fathers conveyed a sense of having made a mistake of unplanned conception with the mothers of their children through a series of bad decisions against their better judgement.

However, Bandile noted:

I was 23 when I had my child, and I was already working. While the pregnancy was unplanned, I felt ready when the child was born because I had bought all the preparation stuff, including clothes. I told the mother of my child not to worry because I was going to look after her during the pregnancy and our child upon birth (Bandile, 24 years old).

The sense of readiness was expressed mostly by young fathers who were permanently employed. There was a strong perception of ‘taking responsibility as a form of masculinity’ among the young fathers who stated that they were ready to be fathers when they discovered that they had conceived a child. In this group of young fathers, the transition into fatherhood was dependent on the willingness to take responsibility for one’s actions for the sake of the mothers of their children and children and not opt out, as many young men do in the face of difficult circumstances.

#### Family influence

Many of the participants in this study indicated negative reactions from their families and the mothers of their children’s families as major factors impeding their roles as partners to the mothers of their children and fathers to their children. These negative reactions from families impacted the young fathers’ involvement prenatally and postnatally. One young father highlighted:

When I first approached my family to tell them that I had impregnated the mother of my child, my mother rebuked me and told me that I was careless. My uncles told me that I needed to focus on my school and leave the mother of my child alone because I was also still a child. They reprimanded me and threatened to chase me out of the family home if they ever saw me with or heard I was with the mother of my child. On the other hand, the family of the mother of my child did not want to hear anything about me. They called me a ‘*Para*’ (a vagrant – with nothing) and did not support my relationship with the mother of my child. Even if I loved her, we ended up breaking up because neither of our families supported us (Sizwe, 19 years old).

These negative reactions shape young fathers’ relationships with the mothers of their children and their children. Most young fathers indicated that while they still loved the mothers of their children and wanted to be present in their children’s lives, their families and those of the mothers of their children made it difficult, leading to eventual separation and non-involvement in their children’s lives.

The few participants who indicated that their families were supportive and enabled them to be involved in their children’s lives highlighted how these positive reactions improved their relationships with the mothers of their children and children. Duduzane noted:

It is easy to be involved in your child’s life if your family, your partner, and their family are supportive. My family has been supportive from the day I told them about my partner’s pregnancy, and they have always encouraged me to be involved in my child’s life. I get along also with my partner. Even if we are not staying together, we do everything for our child together because I also want to be always involved. Sometimes, when my partner is going to the clinic or town alone, she comes to our house and leaves our child with me. Her family is understanding and caring. They are also happy to see me involved in our child’s life (Duduzane, 24 years old).

These sentiments show the importance of social support for young fathers in their transition to fatherhood and how it impacts the involvement of fathers in their children’s lives.

### Context and cultural requirements

This study presents two different contexts: (1) an informal settlement context and (2) a rural context. These two different contexts present different cultural requirements for fathering, family formation, and cohabitation. The participants from the informal settlement highlighted that culture in the context was diluted, and very few families were following the traditional requirements for family formation and cohabitation. When asked about the cultural implications of impregnating the mother of his child, Mzakes noted:

You see here, culturally, it does not really matter whether you impregnated someone or you are staying with the mother of your child without paying *inhlawulo* (acknowledgment of paternity) or *lobola* (bride price). I impregnated the mother of my child, and I still have not gone to talk to the family because the family does not stay here. The family stays in the Eastern Cape. The mother of my child went there when she was about to give birth and came back after birth. I do not stay with her, but I get to see my child every time I want to, even without paying inhlawulo (Mzakes, 23 years old).

These sentiments were in contrast to those participants from the rural community, all of whom highlighted the difficulties of being involved in their children’s lives without paying *inhlawulo*. Zola indicated:

You know, my brother, it is difficult for me even to see my child because the family of the mother of my child says that I first need to go and pay *inhlawulo* for my child. I cannot even spend time with my son because I do not have the money to pay *inhlawulo*. If only they could understand that I want to be involved in my child’s life even when I cannot provide financially, I would be the happiest man, but nowadays everything is about money (Zola, 19 years old).

These shared sentiments highlight the challenges young fathers face in fulfilling cultural expectations of being fathers in rural communities in South Africa.

### Young fatherhood and masculinities

#### Financial provision as a key marker of what makes a man

Young fathers in the study were asked to give their views on what makes a man and fatherhood in South Africa. The most popular response was one about provision as a point of intersection between what makes a man and fatherhood. Most participants stated that their ability to provide financially made them men and involved fathers. Musawenkosi noted:

You cannot be a responsible adult man when you cannot provide financially for yourself and others. You will be a ‘boy’ if you have no means of provision. Even if you are a father and cannot provide for your child, you would not be a proper father. Providing financially is what separates ‘real men’ from ‘boys’. Fatherhood does not only entail being a biological father to a child but also being able to ensure that you provide for that child financially. Money is key to what makes a man and a father (Musawenkosi, 24 years old).

These sentiments indicate how these young fathers associate financial provision with higher masculine status, reinforcing the provider role as a deeply entrenched part of masculine identity. From the study data, it is clear that the young fathers argue that being unable to command financial and material resources undermines men’s involvement in families, both practically and psychologically.

While financial provision was the point of intersection between what makes a man and fatherhood, most participants highlighted the difficulties they were experiencing in meeting this demand. Muzi acknowledged that:

I know that one thing that defines a man and a father is the ability to provide financially. However, this is difficult, particularly for me, because I do not work, and I rely on precarious employment to meet my needs. Sometimes, I cannot even meet my needs. It is not easy being a man because people expect you to provide. It is even more difficult when you have a child, so it is difficult to be an involved father without money (Muzi, 21 years old).

This study highlights that while financial provision was the point of intersection between what makes a man and fatherhood, it was largely multi-determined by external factors beyond individual factors.

#### Present and involved fatherhood as a determinant of what makes a man

Participants in the study also indicated that being a present and involved father defined what makes a man regardless of their resident status with the child. While most participants indicated that caregiving was largely a ‘woman’s responsibility’, they emphasised the importance of being present and involved as fathers. Zweli indicated that:

As a father, if you want to show that you are a ‘real man’, you need to be present and involved in your child’s life. You have to make sure that you are available and easily accessible to your child. You do not need to be co-resident with your child to be present and involved. You need to always be there for them. Your child needs to know that they can come to you for anything they want. You have to show your child love and have a close relationship with them. Your child always needs to be free to talk to you about anything. Even if providing financial support is important, you need to be emotionally available for them all the time (Zweli, 22 years old).

While co-residence is usually associated with the father’s presence, the study participants highlighted the importance of being present and involved beyond residence because none of them were co-residing with their children. The participants also expressed emotional attachment as an important trait for a father to have.

The young fathers in the study reflected on how they grew up without present, caring, and involved fathers and how they wanted a different relationship with their children. The young fathers insisted on creating alternative forms of masculinities. Jabulani indicated that:

I did not learn anything positive from my father. He was absent most of the time, and when I would see him, he was always drunk and abusive to my mother and sisters. I have told myself that I will never be a father like that. I know I am not staying with my child, but I am always present for her when she needs something. I do my best to spend time with her and her mother when we go out. I want to give my child everything that my father never gave me (Jabulani, 23 years old).

This data indicates that the father’s presence and involvement go beyond physical presence and co-residence and that negative childhood experiences can motivate young fathers to be better parents than their fathers by unlearning and transforming the negative experiences they had into positive life choices for themselves and their children.

### Structural factors’ impact on young fathers

This study highlights a number of structural factors that are negatively impacting young fathers, including but not limited to poverty and unemployment, high alcohol and drug abuse, limited access to healthcare facilities, and lack of access to social security services for young fathers.

#### The impact of poverty and unemployment on young fathers

All young fathers in the study stated that they were living in areas of extreme poverty and unemployment and that these contexts made it difficult for them to perform their roles as fathers. Musawenkosi indicated that:

We live in abject poverty and are unable even to get money to go to town and apply for jobs. I cannot even go and fix my CV and print it so that I can have a CV on hand when I am looking for a job. Without money to look for a job, it means I will never be employed, and I will never be able to provide for my child. The conditions in this community are terrible. It is difficult for parents to raise their children when they do not have money to feed them (Musawenkosi, 24 years old).

This data highlights how poverty and unemployment limit opportunities for young fathers, which then trickle down to children. Most young fathers in the study indicated that while their non-co-residence with their children may be misconstrued with reduced father-child involvement, it is actually because of poverty that they cannot afford to pay *inhlawulo* and be able to co-reside with their children. Senzo noted:

As a young father who is also being looked after by my own family, who are struggling to make ends meet, it is difficult for me even to raise money to pay *inhlawulo* for my child. I do not have a permanent job and rely on precarious employment, which does not even pay much. Because I cannot afford to pay *inhlawulo*, I cannot have access to my child. However, it does not mean that I do not love my child. I do, but I do not have the means to provide for her needs (Senzo, 20 years old).

This data shows that the father’s co-residence with his child and involvement is largely dependent on financial resources, which most young fathers in this study do not have access to. This makes it difficult for poor and unemployed young fathers to be co-residents and involved with their children.

#### The impact of high alcohol and drug abuse on young fatherhood

Most unemployed young fathers indicated that high alcohol and drug abuse had negative impacts on their roles as young fathers. On the other hand, employed fathers highlighted regulated alcohol consumption without any drug intake. Scelo, who is unemployed, noted:

You see, I do not have a job, and it is not easy to get one because I do not have a qualification, so I wake up in the morning and go and relax with some of the guys at the Tavern, waiting for anyone to come and buy a beer that we will share until it is finished, then wait for someone else again to buy another beer until it is in the evening then we go home. I cannot go to the mother of my child’s house with R20.00 because it cannot buy anything, so I would rather use it to buy beer and then get other people to buy it for me. The day I get a lot of money, I have to buy for those people who used to buy for me when I had nothing, so it becomes a cycle (Scelo, 23 years old).

Most unemployed young fathers mentioned binge drinking and drug abuse as stress relievers for them. In contexts where employment opportunities are limited to none, young fathers present signs of hopelessness for the future, which leads to alcohol and substance abuse. On the contrary, employment opportunities for young fathers have proved to be a buffer to alcohol and drug abuse. Bandile highlights how he regulates his drinking behaviour so that it does not impede on his work and fatherhood roles.

When I come back from work, I go to check on my child and the mother. Sometimes, I go there to drop off stuff they require, but sometimes, I go to check on them. After that, I go home and change so that I can play snooker and have a beer or two at the Tavern. I do not want to drink too much because I have to save money for my child and also transport to go to and from work every day. I have to drink responsibly because I have responsibilities for my family and for me to protect my work (Bandile, 24 years old).

This finding shows how a sense of one’s responsibilities can protect one from risky behaviour and direct one towards healthy lifestyles for one’s sake and that of one’s children and family.

#### Limited access to healthcare facilities

Participants were asked about the availability of healthcare facilities in their communities and whether they access them for their medical check-ups and treatment. Most participants highlighted that they rarely accessed the community clinics unless they were really sick and needed medical attention. Most participants did not see any association between their healthcare and being a father. Siyabonga noted:

I have never gone to the clinic. Even when I feel sick, I drink water and sometimes sleep it off, but I do not go to the clinic to seek medical attention because the nurses there are very rude. I do not even accompany the mother of my child to the clinic when she goes with the child because I hear the nurses there enjoy shouting at men (Siyabonga, 23 years old).

However, Zweli indicated the importance of knowing one’s health status, not only for themselves but also for their children and family. He highlighted:

You see, when I go to the clinic I am not only doing it for myself but for my child and family. I have to know my health status so that I can make the right choices for myself, my child, and my family. The last time I went to the clinic was three weeks ago to check for my HIV status, Blood Pressure, and Diabetes. I need to be aware so that if there is a disease that needs to be treated, I will attend to it early (Zweli, 22 years old).

The results indicate that most men in the study have poor health-seeking behaviours as they do not access their primary healthcare facilities for medical check-ups or treatment.

## Discussion

This study explored the perceptions of young fathers on young fatherhood, masculinities, and structural factors in South Africa. Consistent with current literature, young fatherhood in this study is associated with a number of structural vulnerabilities, such as living in communities with high alcohol and drug abuse, low educational attainment, unemployment, and poverty (Morrell, 2006; Chili and Maharaj, 2015; Chikovore et al., 2016). The results from this study show that these structural vulnerabilities, which are deeply entrenched in the country’s history and socioeconomic fabric, intersect with prevailing notions of masculinities to influence young men’s pathways to fatherhood negatively.

The findings also highlight that lack of access to resources and the inability to provide financial support influenced both masculine behaviours and fatherhood roles, with fathers mainly construing fatherhood according to the traditional, dominant perceptions by which the most important fatherhood role is to provide financially. These results are consistent with other studies that found the father-provider discourse is often drawn on in communities affected by high poverty and unemployment (Strier, 2014; Enderstein and Boonzaier, 2015). However, this study also established that young fathers acknowledge other forms of fatherhood roles, including active presence and involvement in their children’s lives. These are critical roles for fathers as they form attachments and connections with their children regardless of financial status and provision.

The study results indicate that access to tangible financial and social support resources engendered fatherhood roles. In contrast, the lack of these resources increased the risk of disengagement or non-involvement. Hence, poverty and unemployment in South Africa have created ‘families in waiting’ due to young fathers’ inability to start their own families because of limited access to financial resources. These findings corroborate results from other studies in South Africa (Enderstein and Boonzaier, 2015; Malherbe and Kaminer, 2022; Malinga and Ratele, 2022).

Consistent with my previous research, the impact of family dynamics and support, together with cultural expectations, cannot be understated (Makusha and Richter, 2016). The difficulties in navigating family dynamics and cultural expectations are often huge barriers for fathers, and particularly young fathers to have a relationship with and be involved in their children’s lives. In this regard, there should be a call to put the best interests of the child at the centre. Providing support and encouragement to young fathers to be involved in their children’s lives early on is critical for continuous presence and involvement beyond financial provision.

Lastly, this study found that most young fathers had poor health-seeking behaviour in terms of both not accessing healthcare facilities for medical check-up and treatment and demonstrating high rates of alcohol and drug abuse. These results are consistent with findings elsewhere in the literature indicating that where fathers are living in conditions of distress, poverty, and unemployment, they are trapped in a situation in which they cannot reconcile their roles as fathers and their healthcare, leading to risky behaviours.

### Study limitations

Despite the comprehensive approach and rich qualitative data obtained, this study has several limitations. Firstly, the sample size is relatively small and geographically constrained to specific communities in Durban and Pietermaritzburg, which may limit the generalizability of the findings to other contexts within South Africa or beyond. Additionally, the use of purposive and snowball sampling techniques might have introduced selection bias, as participants who are more visible or willing to share their experiences may differ in significant ways from those who are less accessible or reluctant. Furthermore, the reliance on self-reported data can be subject to social desirability bias, where participants may present themselves in a more favourable light, particularly regarding sensitive topics like fatherhood and masculinities. The study’s cross-sectional design also limits the ability to draw conclusions about the causal relationships between structural factors, masculinities, and fatherhood experiences. Finally, while interviews and focus groups were conducted in Zulu and later translated into English, some nuances of language and meaning may have been lost in translation, potentially affecting the depth and accuracy of the data interpretation.

## Conclusion

In South Africa, young men’s involvement during the transition to fatherhood appears multi-determined. The nexus of structural factors, masculinities, and young fatherhood underscores the complex interplay of socioeconomic, cultural, and historical forces shaping men’s experiences as fathers. Furthermore, the stigma surrounding young fatherhood, coupled with punitive attitudes towards adolescent sexuality, often hinders young men’s access to reproductive health services and parental support programmes. Addressing these barriers requires a comprehensive approach that acknowledges the intersecting influences of structural factors and masculinities on young fatherhood while promoting gender-equitable norms and access to resources. To effectively support young fathers and promote family well-being, it is imperative to address the root causes of structural inequalities, challenge rigid norms of masculinities, and foster inclusive policies and programmes that empower young men to embrace their roles as caregivers and agents of change within their families and communities. Only through such holistic interventions can South Africa strive towards a more equitable and just society for all its citizens, regardless of age, gender, or socioeconomic status.

## Data availability statement

The raw data supporting the conclusions of this article will be made available by the authors, without undue reservation.

## Ethics statement

The studies involving humans were approved by the Human Sciences Research Council’s Research Ethics Committee. The studies were conducted in accordance with the local legislation and institutional requirements. The participants provided their written informed consent to participate in this study.

## Author contributions

TM: Conceptualization, Data curation, Formal analysis, Funding acquisition, Investigation, Methodology, Project administration, Resources, Software, Supervision, Validation, Visualization, Writing – original draft, Writing – review & editing.
